# Genome-Wide Analysis of *KNOX* Genes: Identification, Evolution, Comparative Genomics, Expression Dynamics, and Sub-Cellular Localization in *Brassica napus*

**DOI:** 10.3390/plants14142167

**Published:** 2025-07-14

**Authors:** Xiaoli He, Ruiyi Zheng, Yan Chen, Chengfang Tan

**Affiliations:** 1 College of Landscape Architecture and Art Design, Hunan Agricultural University, Changsha 410128, China; xiaolihe2000@163.com; 2 Orient Science & Technology College, Hunan Agricultural University, Changsha 410128, China; 13607509726@163.com (R.Z.); 16684039195@163.com (Y.C.); 3 College of Bioscience and Biotechnology, Hunan Agricultural University, Changsha 410128, China; 4 Key Laboratory of Crop Epigenetic Regulation and Development in Hunan Province, Changsha 410128, China; 5 Yuelu Mountain Laboratory, Changsha 410128, China

**Keywords:** *Brassica napus* L., Genome-wide analysis, KNOX, *Brassicaceae*

## Abstract

*KNOX* genes play crucial roles in cell-fate determination and body plan specification during early embryogenesis. However, the specific gene structure and functional differentiation of *KNOXs* in *Brassica napus* is still unclear. We investigated *KNOX* genes in *Brassica rapa* (*B. rapa*), *Brassica oleracea* (*B. oleracea*), and *Brassica napus* (*B. napus*), which are polyploidy models with genome triplication after *Arabidopsis*-*Brassiceae* divergence. In total, 15, 14, and 32 *KNOX* genes were identified in *B. rapa*, *B. oleracea*, and *B. napus*, respectively. Phylogenetic analysis classified *BnKNOXs* (*B. napus*) into three classes with conserved domain organization. Synteny analysis indicated that *BnKNOXs* family expansion during allopolyploidization was mainly due to whole-gene and segmental duplications. *Cis*-element, gene structure, and expression pattern analyses showed high conservation within the same group. RNA-seq and qRT-PCR results divided *BnKNOXs* into three classes with distinct expression patterns: Class I exhibited moderate and specific expression in buds and inflorescence tips; Class III showed specific low expression in seeds and stamens; while the second class showed expression in most tissues. Sub-cellular localization results showed that the three candidate genes from the three classes exhibited distinct subcellular localizations, with BnSTM-C and BnKNAT3a-A predominantly in the nucleus and BnKNATM1-A in the cytoplasm indicating different expression patterns. Collectively, these findings provide a foundation for further functional studies of *BnKNOX* genes in *B. napus*.

## 1. Introduction

Homeotic genes encode proteins that include a conserved 60-amino-acid homeodomain, which is essential for regulating the expression levels of target genes and thus plays a pivotal role in the developmental processes of organisms [1,2]. These genes were first identified in fruit flies and found to be involved in segmental development as members of the *Bithorax* and *Antennapedia* complexes [3,4]. In the plant kingdom, maize *Knotted-1* (*Kn1*) was the first identified gene that encodes a homeodomain-containing protein. Subsequently, *KNOX* genes have been cloned from a diverse array of plant species, revealing an expansion in the membership of the *KNOX* gene family in conjunction with the evolutionary development of multicellular diploid plants [5,6]. Unicellular green algae and red algae harbor a solitary *KNOX* gene, whereas in terrestrial plants, KNOX proteins are commonly encoded by a complement of genes within diverse gene families [7]. Plant homeotic genes are classified based on their sequence differentiation and the fusion with characteristic codomain sequences [8,9,10], resulting in their division into 14 distinct classes [11]. Knotted-like homeobox (KNOX) belongs to the three-amino acid loop extension super class, which is characterized by three additional residues between helixes 1 and 2, and it is regarded as one of the oldest homeobox gene classes [12,13,14].

Based on sequence similarity, structural characteristics, phylogenetic relationships, and expression patterns, *KNOX* genes are mainly divided into three classes [15,16,17], and they have been found in almost all plant species [6,18]. Class I and II KNOX proteins always contain KNOX1, KNOX2, ELK, and homeobox_KN domains [19]. The two KNOX domains, located at the N-terminus, play an important role in phenotypic change in transgenic plants [20,21]. The ELK domain encodes nuclear localization signals and facilitates interaction with other proteins as well as transcriptional inhibition [21]. The HD domain is located at the C-terminal and it is involved in DNA binding and homodimer formation [22]. KNOX subfamily in Class III lacks the ELK-HD domain. *KNOX* genes play a crucial role in regulating plant growth and development by controlling meristem and vascular cambium activity in the stem [23], axillary bud [24], leaf primordial [25], and gametophyte development [26,27].

In single-celled green algae (*Chlamydomonas reinhardtii*), the primary function of the KNOX protein is to regulate the sex of the gametophyte and the development of the fertilized egg [27]. In bryophytes (*Physcomitrella patens*), although the two *KNOX* genes are mainly expressed in sporophytes, the KNOX proteins encoded by them are significantly differentiated in function [28]. Class I KNOX is involved in promoting cell proliferation during the sporophyte development without inducing shoot formation of the moss *Physcomitrella patens*, while Class II KNOX protein inhibits gametophyte development [29]. KNOX in class I is essential for sporophyte meristem development in vascular plants but dispensable for gametophyte development in ferns [30]. In *Arabidopsis thaliana*, four Class I *KNOX* genes, including *SHOOTMERISTEMLESS* (*STM*, AT1G62360), *KNAT1* (AT4G08150), *KNAT2* (AT1G70510), and *KNAT6* (AT1G23380), play crucial roles in the maintenance and function of the shoot apical meristem (SAM) [25,31]. Cytokinin can induce the expression levels of *STM* and *KNAT1* in SAM. In addition, *AtSTM* was proved to be associated with carpel and inflorescence meristem development [22,32]. AtKNAT1 targets TCP15 to modulate filament elongation during stamen development [33]. KNAT6 and STM exhibit functional redundancy in the upkeep of the vegetative SAM, with KNAT6 being positioned at the regulatory boundaries within embryos through the *STM/CUC2* (*cup-shaped cotyledon 2*) pathway [32]. Class II *KNOX* genes, including *KNAT3* (AT5G25220), *KNAT4* (AT5G11060), *KNAT5* (AT4G32040), and *KNAT7* (AT1G62990), exhibit diverse expression patterns [34,35,36]. Class II *KNOX* genes exhibit expression across various angiosperm organs, including roots, stems, leaves, and flowers, playing a pivotal role in the orchestration of plant organ differentiation. Furthermore, these genes are implicated in the negative regulation of secondary cell wall synthesis in the vascular bundle fibers of the inflorescence stem [37]. For instance, KNAT3 and KNAT7 play multiple roles in the development of secondary cell walls and redundantly regulate mucilage biosynthesis in *Arabidopsis* seeds [38]. *Arabidopsis* KNATM, which is defined as a novel *KNOX* transcriptional regulator, encodes a homeodomain protein expressed in both reproductive and vegetative meristems. It is also involved in leaf proximal-distal patterning [17], and its homologs belong to a new class [39]. Class I and Class II KNOX proteins, acting as transcription factors, share downstream targets but exert opposite effects: while Class I KNOX proteins activate target genes, Class II KNOX proteins negatively regulate these genes [7].

*KNOX* genes regulate plant development, with their role primarily elucidated in Arabidopsis [25,33]. Although extensive studies have identified *KNOX* genes in various species [30], limited knowledge exists regarding *Brassica* species. *B. napus* is an allotetraploid (*AACC*, 2n = 38) oilseed species providing almost 15% of vegetable oil worldwide with a complex genome, which was formed by natural hybridization between the two diploid species *B. rapa* (*AA*, 2n = 20) and *B. oleracea* (*CC*, 2n = 18). Genome-wide duplications and chromosomal rearrangements are pivotal in propelling the diversification and evolutionary processes of plant species. Consequently, the three Brassica species serve as an exemplary model for investigating the impact of extensive chromosomal modifications on genomic evolution following the divergence from *Arabidopsis* to the *Brassicaceae* family [40]. However, a comprehensive investigation of the *KNOX* gene family in *Brassicaceae*, especially in *B. napus*, remains unexplored. In this study, we identify and phylogenetically analyze the *KNOX* genes in *Brassica* species. Subsequently, we analyze the structures, *cis*-elements, and the expression patterns of the KNOX gene family, as well as sub-cellular localization in *B. napus*. This work provides new insights into the evolutionary significance of functional differentiation of these genes and lays a foundation for future research on *KNOX* genes in *B. napus*.

## 2. Results

### 2.1. Identification and Chromosome Map of KNOX Proteins and Genes from Brassica

A total of 15 KNOX proteins from *B. rapa*, 14 from *B. oleracea*, and 32 from *B. napus* were identified on the official website (BRAD (brassicadb.cn)), while an additional 89 members were screened from the genomes of several green lineage species (Phytozome (Phytozome), version 13.0) (Supplement File S1). KNOX proteins are exclusively found in higher plants, except one member with the KNOX2 domain identified in *Ostreococcus lucimarinus*; they cannot be detected in lower plants such as *Chlamydomonas reinhardtii*, *Volvox carteri*, and *Phaeodactylum tricornutum*, despite having HOX homologous (Supplement File S1).

**Figure 1:**
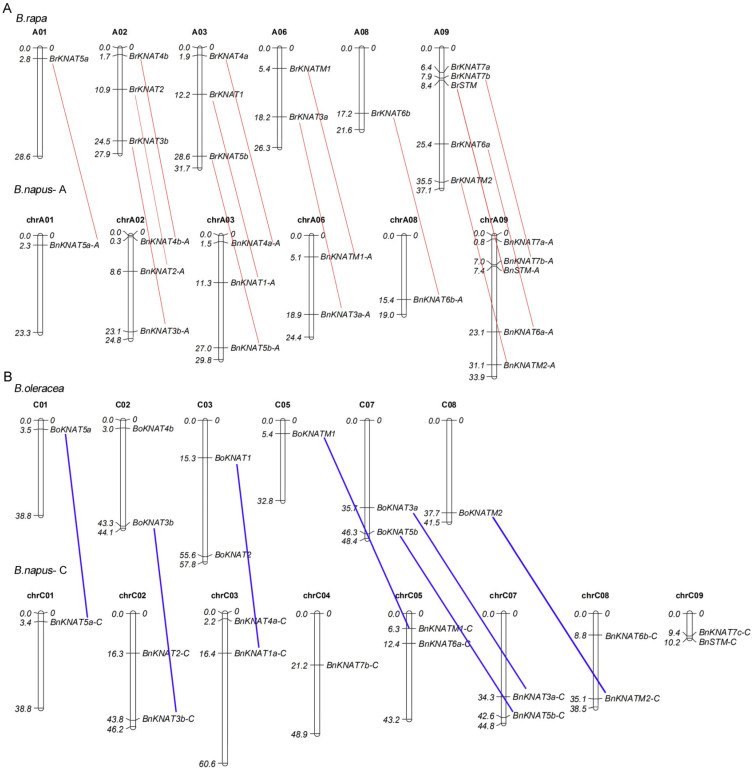
Chromosomal distribution of *KNOX* genes from *Brassica.* (**A**): A-genome of *B. rapa* and *B. napus*. A01–A06, A08, and A09 are the chromosomes from the A-genome of *B. rapa*; chrA01–A06, chrA08 and chr are the chromosomes from the A-genome of *B. napus*; (**B**): C-genome of *B. rapa* and *B. napus*. C01–C03, C05, C07, and C08 are the chromosomes from the C-genome of *B. oleracea*; ChrC01–C06 and C07–C09 are the chromosomes from the C-genome of *B. napus*. The marks on the left represent the chromosome sizes. The red and blue lines show the syntenic gene pair. The syntenic genes of *BoSTM*, *BoKNAT4a*, *BoKNAT6a/6b*, and *BoKNAT7* are not shown due to their uncertain locus on chromosome. The red and blue lines show the syntenic gene pairs between *B. napus* and *B. rapa* or *B. oleracea*.

The *KNOX* genes of *B. rapa* were mapped onto six chromosomes. Chromosome A09 showed the highest number of five *KNOX* genes, followed by chromosome A02/03 with three genes. Chromosomes A04, A07, and A10 did not contain any *KNOX* genes. Nine *KNOX* genes of *B. oleracea* were also mapped onto six chromosomes (C01-03, C05, C07, and C08), while *BoSTM*, *BoKNAT4a*, *BoKNAT6a/6b*, and *BoKNAT7* could not be successfully mapped. In total, 29 *KNOX* genes of *B. napus* were mapped onto 14 chromosomes (six A-chromosomes and eight C-chromosomes). However, three C-genome specific genes (*BnKNAT1b-C*, *BnKNAT4b-C* and *BnKNAT7a-C*) could not be mapped (Figure 1).

### 2.2. Phylogenetic and Classification Analyses of KNOX Protein Family

**Figure 2:**
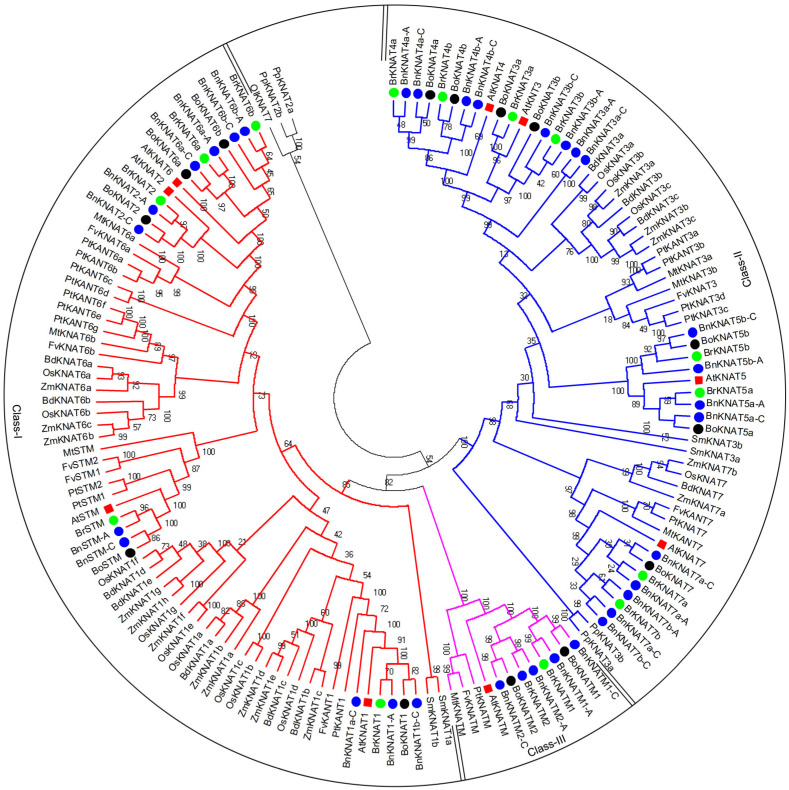
Phylogenetic tree of plant KNOX proteins. This tree includes 150 KNOX proteins from *Brassica napus* (32), *Populus trichocarpa* (16), *Zea mays* (16), *Brassica rapa* (15), *Brassica oleracea* (14), *Oryza sativa* (13), *Brachypodium distachyon* (11), *Arabidopsis thaliana* (9), *Fragaria vesca* (8), *Medicago truncatula* (7), *Physcomitrella patens* (4), *Selaginella moellendorffii* (4), and *Ostreococcus lucimarinus* (1) genomes. *Chlamydomonas reinhardtii*, *Volvox carteri*, and *Phaeodactylum tricornutum* are not analyzed because of lacking KNOX domain proteins. The proteins were selected for the phylogenetic tree based on their homology to *Arabidopsis*. The KNOX proteins can be grouped into three classes, Class I-III, based on the phylogenetic tree and domain organization. PpKNAT2a/2b and OlKNAT6 are not included in any class and will not be discussed in this paper.

- To analyze the evolutionary patterns of KNOX, we constructed a comprehensive phylogenetic tree for 150 KNOX proteins, including the following: 32 from *B. napus*, 16 from *Populus trichocarpa*/*Zea mays*, 15 from *B. rapa*, 14 from *B. oleracea*, 13 from *Oryza sativa*, 11 from *Brachypodium distachyon*, 9 from *Arabidopsis thaliana*, 8 from *Fragaria vesca*, 7 from *Medicago truncatula*, 4 from *Physcomitrella patens*/*Selaginella moellendorffii*, and 1 from *O. lucimarinus* (Figure 2; Supplement File S1). Subsequently, excluding PpKNAT2a/2b and OlKNAT7 due to their inability to be classified into Class I or Class II despite sharing the same domain organization as others (similar results were obtained using different phylogenetic trees constructed by Neighbor-Joining or Maximum Likelihood methods), we successfully categorized the remaining set of 147 KNOX proteins into three distinct classes. Notably in *Brassica* species specifically, *B. napus* not only encompasses all KNOX proteins found in both *B. rapa* and *B. oleracea* but also exhibits an additional three unique members within its repertoire (Figure 2; Supplement File S1).

#### 2.2.1. Phylogenetic and Domain Analyses of Class I

**Figure 3:**
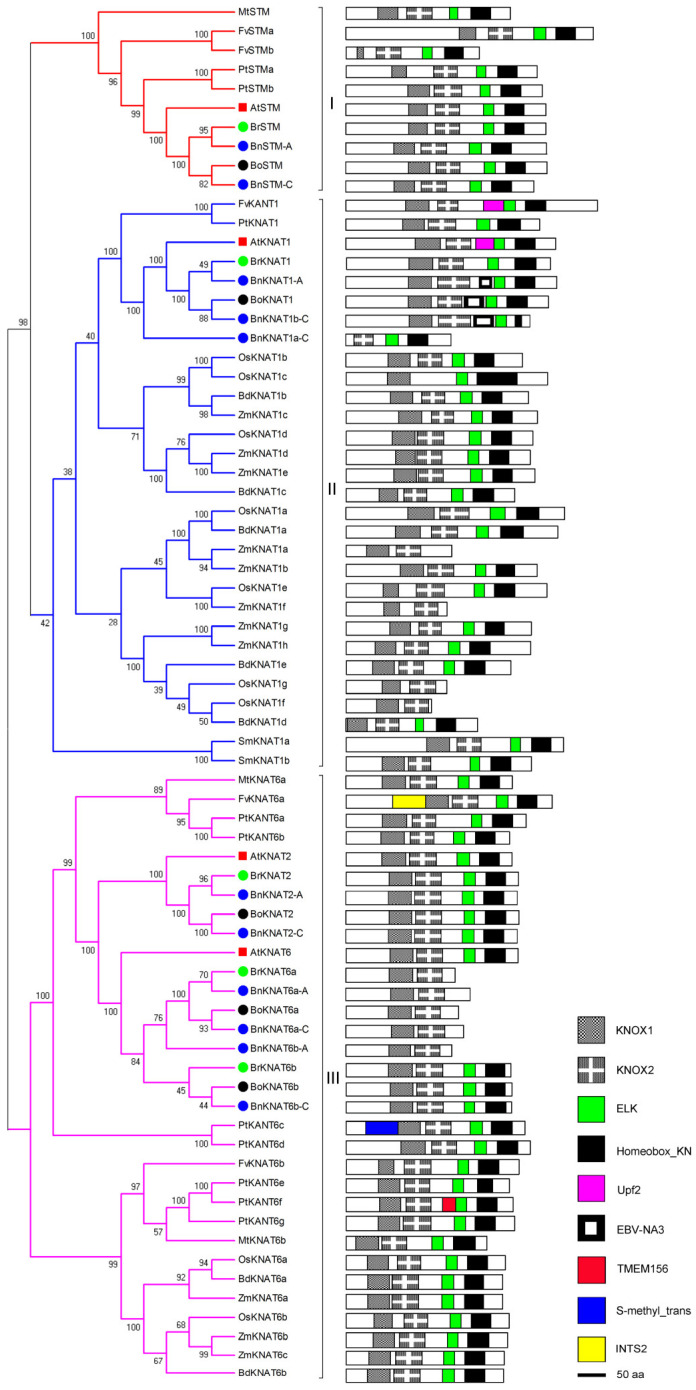
Phylogenetic and domain analyses of Class I. Monocotyledons have more copies of KNAT1, but no STM members. *B. rapa* and *B. oleracea* have homologous proteins to those in *Arabidopsis* Class I, and two homologs of AtKNAT6. *B. napus* includes both KNOX proteins of *B. rapa* and *B. oleracea*, but two copies of BoKNAT7. Domain organizations of homologous proteins are conserved.

*Arabidopsis* Class I KNOX genes play a crucial role in shoot apical meristem (SAM) activity, carpel development, sporophyte development, and abscission zone development [41]. Class I specifically encompasses KNOX proteins found in vascular plants, including 2 members in *S. moellendorffii*, 3 in *M. truncatula*, 4 in *A. thaliana*, 5 in *B. rapa*/*B. oleracea*/*F. vesca*, 7 in *B. distachyon*, 9 in *P. trichocarpa*/*O. sativa*, 10 in *B. napus*, and 11 in *Z. mays* (Figure 3; Supplement File S1). Monocots exhibit a higher number of AtKNAT1 homologous proteins compared to dicots, while maintaining highly conserved domain organizations characterized by the major domains: KNOX1 (PF03790), KNOX2 (PF03791), ELK (PF03789), and Homeobox (PF00046). The phylogenetic relationships of the protein classes are closely linked to plant species evolution (Figure 3). Within Class I, KNOX genes can be categorized into three branches labeled as Groups I, II, and III. Group I represents the STM group with a conserved domain organization. Group II KNAT1 comprises the largest group. Most species including maize and rice possess more than two copies of KNAT1. Notably, the Up-frameshift suppressor 2 (Upf2) domain is identified within AtKNAT1 and FvKNAT1 [42]. The Upf2 domain is conserved in eukaryotes and is crucial for mRNA decay [43]. Epstein–Barr virus nuclear antigen 3 (EBNA-3, PF05009), which is an EBNA family member that responds to stimulated Epstein–Barr virus-specific T cells during adoptive immunotherapy is found in BoKNAT1 [44]. However, OsKNAT1f/1g and BdKNAT1d lack the ELK and Homeobox domains, respectively. Group III includes AtKNAT2/AtKNAT6 and its homologs. In addition to the major domains, FvKNAT6a contains an Integrator complex subunit 2 (INTS2, PF14750) domain, that is involved in snRNA transcription and processing. PtKANT6c contains an S-methyl trans domain (Homocysteine S-methyltransferase, pfam02574), and PtKANT6f contains a functionally unknown TMEM156 domain. Fern has similar domain organizations (Figure 3).

In Class I, both *B. rapa* and *B. oleracea* exhibit a total of five members, including two homologs of AtKNAT6 as well as one homolog each of AtSTM, AtKNAT1, and AtKNAT2 (Figure 3; Supplement File S2). The domain organizations remain highly conserved except for the duplicated BrKNAT1 members BrKNAT6a/BoKNAT6a which lack ELK and Homeobox domains [17] and are not detected among the Group III proteins that also play roles in KNOX transcriptional regulation and leaf proximal-distal patterning.

*B. napus* contains 11 homologous proteins (BoKNAT1 duplication) of *B. rapa* and *B. olereace*. The domain organizations of *B. napus* KNOX proteins exhibit a high degree of conservation with their respective donors. Notably, BnKNAT1a-A shows an increased EBV-NA3 domain compared to BrKNAT1, while BnKNAT6b-A lacks the ELK and Homeobox found in BrKNAT6b. Some sequence lengths also exhibit variability, such as BnSTM-C compared to BoSTM and BnKNAT6a-A relative to BrKNAT6, Figure 3). All Class I *KNOX* genes of the three *Brassica* species are syntenic to corresponding genes in *Arabidopsis*, except BnKNAT1b-C and BnKNAT6a-C (Supplement File S2). The syntenic genes suggest existence of the orthologs between *B. napus* and its parental species, namely, *B. rapa* and *B. oleracea*. In addition to inheriting most *KNOX* genes from its parents, new *KNOX* genes are also present in the genome of *B. napus* (*BnKNAT1b-C*, *BnKNAT4b-C*, *BnKNAT6a-C*, *BnKNAT7a-A*, *BnKNAT7a-C*, *BnKNAT7c-C*), while the BoKNAT6a ortholog is lost.

#### 2.2.2. Phylogenetic and Domain Analyses of Class II

**Figure 4:**
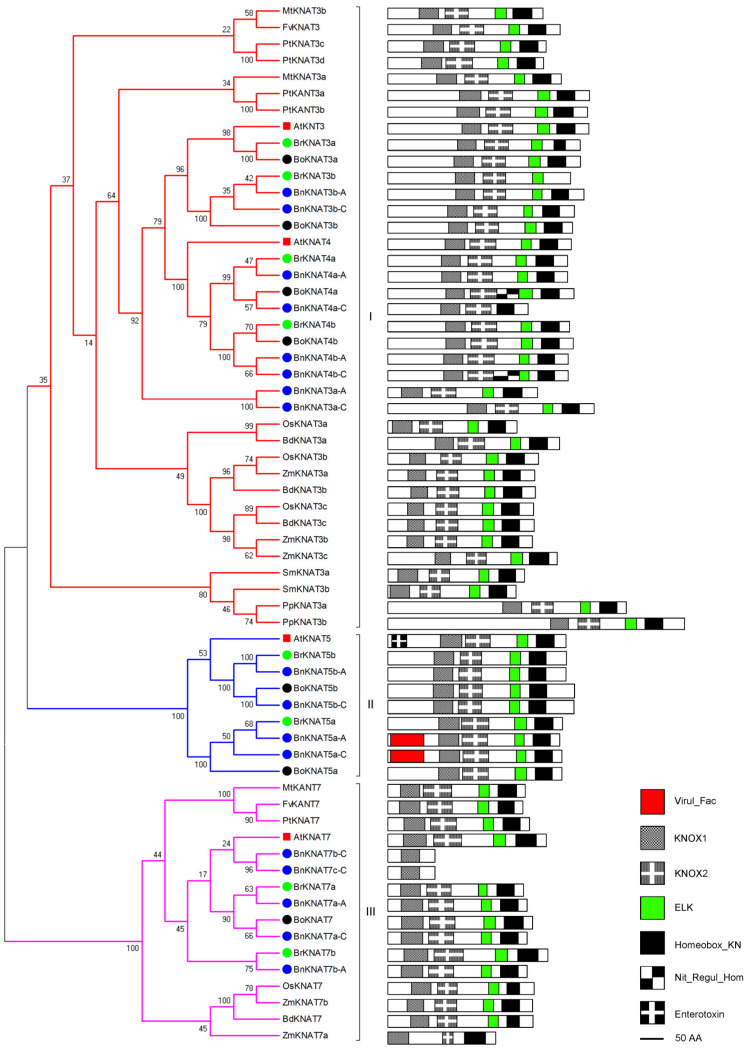
Phylogenetic and domain analyses of Class II. Dicots have more members than monocots. *B. rapa* and *B. oleracea* contain duplicated conserved copies of almost all KNOX proteins. *B. napus* includes both KNOX proteins of *B. rapa* and *B. oleracea*, but three copies of BoKNAT7, and domain organization are similar to their donors.

The functions of Class-II *KNOX* genes remain unclear, but they are potentially involved in the regulation of tissue differentiation, seed germination, root development and secondary wall formation [45,46]. Class-II may contain older KNOX proteins, and can be detected in all higher plants. A total of 2 members are present in *P. patens*/*S. moellendorffii*/*F. vesca*, 3 in *M. truncatula*, 4 in *A. thaliana*/*B. distachyon*/*O. sativa*, 6 in *Z. mays*/*P. trichocarpa*, 7 in *B. oleracea*, 8 in *B. rapa* and 17 in *B. napus* (three copies of BoKNAT7) (Figure 4; Supplement File S1). Similar to Class I *KNOX* genes, the major domains found within Class II include KNOX1, KNOX2, ELK and Homeobox domain. The phylogenetic tree reveals three distinct groups: Group I consists of KNAT3/KNAT4, Group II contains KNAT5 and Group III includes KNAT7. KNOX proteins of fern and moss are at the root of Group-I. Group-I contains multiple members and two or three homologous proteins from each organism except for the eight members from *B. napus*, four from *P. trichocarpa* and one from strawberry. The phylogenetic relationships among Group I proteins are related to plant species evolution (Figure 4). The domain organizations within Group-I are conserved except for BoKNAT4a and BnKNAT4a-C/4b-C. Group II exclusively comprises Cruciferae. AtKNAT5 possesses an additional enterotoxin motif in the heat-labile enterotoxin alpha chain. BnKNAT5a-A/5a-C have additional Virul-Fac motif. Group III encompasses AtKNAT7 and its homologs with conserved domain organizations except for ZmKNAT7a, which lacks the ELK domain, and BnKNA7a-C/7b-C which only possesses the KNOX1 domains.

All *KNOX* genes are duplicated in *Brassica*, with the exception of *B. oleracea* due to the presence of only one copy of *KNAT7* in Class-II. Duplicated genes of *Brassica* encode proteins with conserved domain organizations. Based on the conserved relationships with *Arabidopsis* homologs, it is suggested that *BrKNAT3a*/*3b* and *BoKNAT3a/3b* may be involved in seed germination and early seedling development, while *BrKNAT7a/7b* and *BoKNAT7* play roles in secondary wall formation.

*B. napus* has more than two of the sums of *B. rapa* and *B. oleracea* and the domain organizations are much conserved with their donors, except for BnKNAT4a-C lacking the ELK domain, BnKNAT7b-C/7c-C containing only the KNOX1 domain, and BnKNAT5a-A/5a-C carrying an additional Virul-Fac domain (pfam10139, Figure 4). Similar to Class I genes, all Class II genes of *B. rapa* and *B. oleracea* show synteny with *Arabidopsis* homologs except for *BnKNAT4b-C/7a-A/7a-C/7c-C* (Supplement File S2).

#### 2.2.3. Phylogenetic and Domain Analyses of Class-III

**Figure 5:**
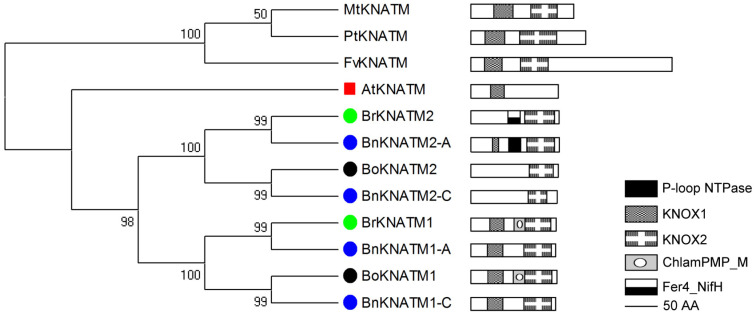
Phylogenetic and domain analyses of Class III. This class is only found in dicots. *B. rapa* and *B. oleracea* contain two copies of AtKNOX. *B. napus* includes both KNOX proteins of *B. rapa* and *B. oleracea*. Domain organizations of homologous KNOX of three *Brassica* species are diverse.

The KNATM, a novel KNOX subfamily, is maintained by a homeodomain-independent mechanism [17,19]. A bioinformatic analysis shows that KNATM is found only in dicots and that it lacks the ELK and Homeobox domain (Figure 5; Supplement File S1). Class III contains only one member each in *F. vesca*, *M. truncatula*, *A. thaliana* and *P. trichocarpa, B. rapa* and *B. oleracea* contain duplicated KNATMs, whereas *B. napus* has quadruple KNATMs.

The relationships of Class III proteins are related to plant evolution but domain organizations are not well conserved (Figure 5). PtKNATM, FvKNATM and MtKNATM conserve KNOX1 and KNOX2 domain organizations. AtKNATM only possesses the KNOX1 domain, whereas BoKNATM2 and BnKNAT2-C contain the KNOX2 domain. BrKNATM2 has KNOX2 and a Fer4_NifH domain (PF00142), which is found in various proteins that share a common ATP-binding domain. Conversely, BnKNATM2-A displays typical KNOX1, KNOX2 and P-loop NTPase domains. In addition to the KNOX1 and KNOX2 domains, BrKNATM1 and BoKNATM1 have an additional Chlamydia polymorphic membrane protein middle (ChlamPMP_M) domain (PF07548). However, their homologs BnKNATM1-A/1-C in *B. napus* lack these domains (Figure 5). Similar to Class II genes, all genes in Class III from *B. rapa* and *B. oleracea* show synteny with *Arabidopsis* homologs (Supplement File S2).

### 2.3. Analysis of Cis-Acting Elements of BnKNOX Gene Promoters and Gene Structure in B. napus

**Figure 6:**
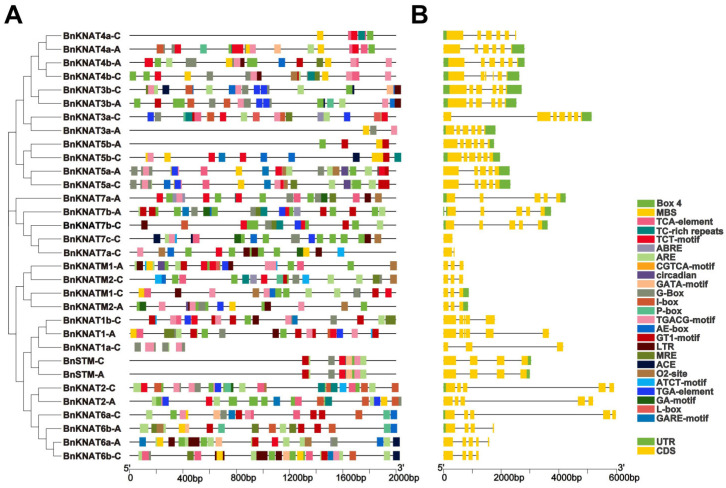
*Cis*-elements and gene structure analysis of *BnKNOXs* family in *B. napus*. (**A**): Different *cis*-elements of *BnKNOXs*. The elements existed in the 2 kb upstream region of *BnKNOXs*, displayed in differently colored boxes. The core *cis*-acting elements CAAT and TATA boxes were identified in all promoters, not shown in the figure. (**B**): The exon-intron structure of *BnKNOX* genes. Exons, introns, and UTRs are indicated by yellow boxes, black lines and green boxes, respectively. The length of genes can be represented using the scale at the bottom.

The *cis*-acting elements of promoters specifically bind to transcription factors to form transcription initiation complexes, which initiate gene expression. Therefore, we identified 717 *cis*-elements belonging to 26 different types (Figure 6). These *cis*-elements could be divided into three groups, plant growth and development, phytohormone responses and abiotic stress responses. For instance, we detected 12 light-responsive *cis*-elements involved in growth and development with a cumulative occurrence of 388: ACE, AE-box, ATCT-motif, Box 4, GA-motif, GATA-motif, G-Box, GT1-motif, I-box, L-box, MRE and TCT-motif (Supplement Files S8). Among the *cis*-acting elements involved in hormone response, ABRE, GAREs (GARE-motif and P-box), O2-site, TCA-element, TGA-element and the MeJA-responsive (CGTCA-motif and TGACG-motif) were identified in the promoter elements regions of 59, 18, 14, 28, 18 and 98 occurrences respectively. Additionally, drought and low temperature-stress related *cis*-acting elements were also detected in the promoter regions of *BnKNOX* genes. These findings indicate that *BnKNOXs* may be pivotal in modulating the growth of plants development and may help elucidate precise functions of the proteins from the *BnKNOX* genes family.

In order to explore the structural diversity of *BnKNOXs*, a comparative analysis was conducted on the gene structures. Visual analysis revealed that while most family members exhibit similar counts of exons and introns, there is variation in their length. Furthermore, it was observed that the majority of these members possess 3–7 exons, with the exception of *BnKNAT7a-C* which contains 2 exons, and the *BnKNAT7c-C* gene which contains only 1 exon. The distribution pattern of exons and introns appears to be intricate, suggesting a potential correlation with phylogenetic subgrouping.

### 2.4. Gene Collinearity and Duplication of BnKNOXs in B. napus

**Figure 7:**
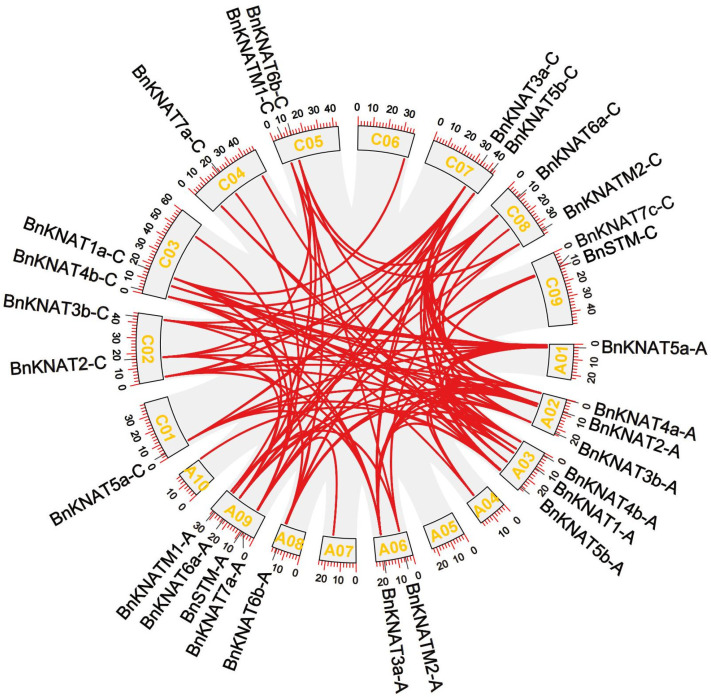
Gene collinearity and duplication of *BnKNOXs* in *B. napus*.

The gene collinearity analysis facilitates the discovery of homologous sequences within species, which can be used as evidence of the whole genome duplication events. We detected 26 duplication events, 15 of which occurred between subgenome A and C (Figure 7). Notably, the *BnKNOXs* on chromosome A05 were not collinear with BnKNOX genes on other chromosomes (Figure 7, Supplement File S4). In chromosomes, gene family can expand by tandem, segmental replication or whole genome [47]. In *BnKNOXs* gene family, 24 pairs were amplified by fragment replication, and only 1 pair (*BnKNAT7b-A/BnKNAT7a-A*) were amplified by tandem replication (Supplement File S5). This result suggested that fragment replication events contributed most to the expansion of the *BnKNOXs* in *B. napus*. Previous studies show that duplication of genes can prevent the loss of function caused by genetic mutation [48,49].

To analyse the mechanism by which *BnKNOX* gene family evolved, the Ka/Ks ratio was calculated on the 26 pairs of genes with collinearity. The results showed that the Ka/Ks ratio of all duplicated *BnKNOX* gene pairs were <1 (Supplementary File S4), which indicates that this family was subject to purifying negative selection throughout evolution. Additionally, the duplication events date was estimated around 0.5–31 MYA (Million Years Ago).

Inter-chromosomal relationship of the *BnKNOXs* in *B. napus* genome. The red lines show the syntenic blocks, while the gray lines collinear blocks in the whole genome.

### 2.5. Expression Patterns of BnKNOXs in Different Tissues from BrassicaEDB

**Figure 8:**
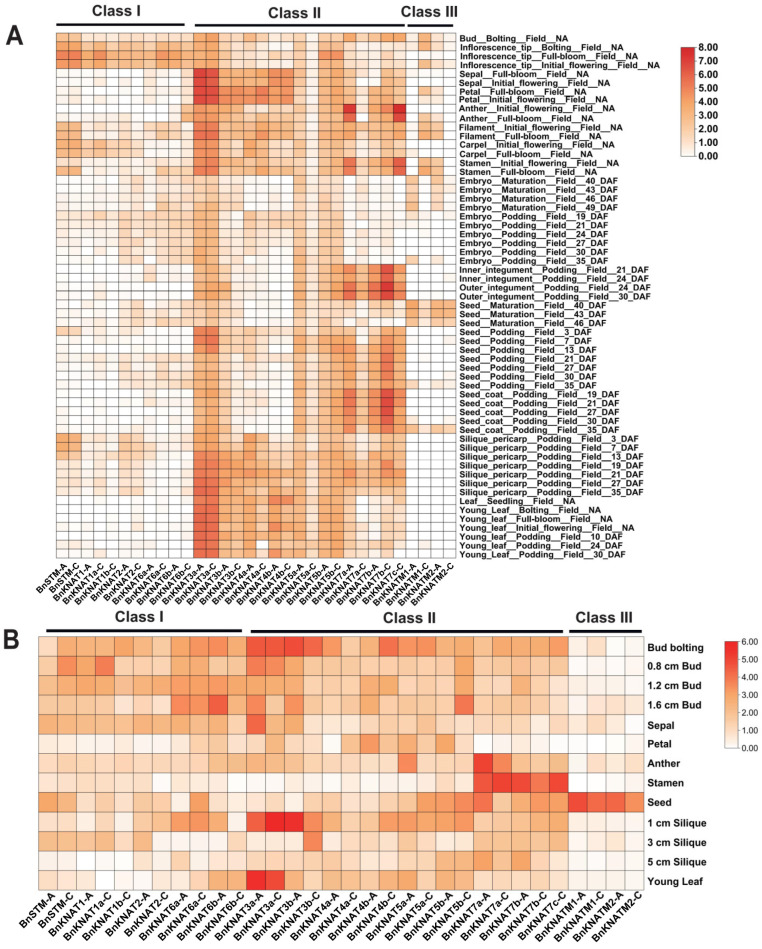
Tissue-specific expression pattern of *BnKNOXs* genes. (**A**): The heat map of relative expression was generated using TBtools software. The data was collected from BrassicaEDB and normalized by log_2_FPKM transformed. (**B**): *BnKNOXs* Expression levels in reproductive organs by qRT-PCR.

The expression patterns of a gene are closely related to its function. For a comprehensive understanding of *BnKNOXs* functions, we analyzed all members’ expression patterns during flowering and fruiting transition stage using available transcriptome data in different tissues including young leaves, roots, seeds, flowers, siliques (Figure 8A). The results showed that all *BnKNOX* genes were expressed in at least 1 tissue indicating they may play a vital role during the developmental stage. Remarkably, the expression pattern of *BnKNOXs* in different tissues was quite different among the three subgroups, while the members clustered in the same group showed a relatively similar expression pattern (Figure 8A). It means that *BnKNOXs* may affect diverse biological functions in different tissues. The transcripts of most genes from group II were relatively highly observed in most of the tissues, reflecting their ubiquitous roles in plant development. The four *BnKNOXs* (*BnKNATM1-A*, *BnKNATM1-C*, *BnKNATM2-A* and *BnKNATM2-C*) in group III were expressed at very low levels in all tissues except for higher expression in mature seed coat.

Similarly, *BnKNOXs* from group I were concentratedly expressed only in stem, root, and inflorescence tip. For example, *BnSTM-A*, *BnSTM-C* and *BnKNAT1a-C* were highly expressed in various stages of stem, suggesting their important roles in stem development.

### 2.6. BnKNOXs Expression Levels of in Reproductive Organs by qRT-PCR

Numerous studies have demonstrated the significant roles of KNOX genes in floral organs [50] and fruit development [51]. To confirm the temporal and spatial expression patterns of BnKNOX genes, we subsequently conducted qRT-PCR experiments in various tissues, including buds (bolting bud, 0.8 cm bud, 1.2 cm bud, 1.6 cm bud), floral organs (sepal, petal, anther and stamen), seeds, siliques (1 cm silique, 3 cm silique and 5 cm silique) and young leaves (Figure 8B). The expression level of BnSTM-A in young leaves was used as a reference with a value set at 1. Overall, the qRT-PCR results are consistent with the transcriptome data trends observed. For example, the expression profile showed that Class I and Class II *KNOX* genes were expressed broadly in the bud development compared with Class III group. Notably, *BnKNAT3a-A* and *BnKNAT3a-C* displayed high expression levels in bolting buds, suggesting their potential involvement in inflorescence formation. Furthermore, we found significantly elevated expressions of five BnKNOXs (*BnKNAT7a-A*, *BnKNAT7a-C*, *BnKNAT7b-A*, *BnKNAT7b-C* and *BnKNAT7c-C*) specifically in stigma indicating their putative roles in stigma development. Interestingly, *BnKNOXs* in Class III (*BnKNATM1-A*, *BnKNATM1-C*, *BnKNATM2-A*, *BnKNATM2-C*) were specifically highly expressed in seeds, implying their crucial functions during seed development processes. Additionally, *BnKNAT3a-C* and *BnKNAT3b-A* might play vital roles during silique development stage. Moreover, gene members belonging to the same group exhibited similar expression characteristics.

### 2.7. Subcellular Localization Analysis

**Figure 9:**
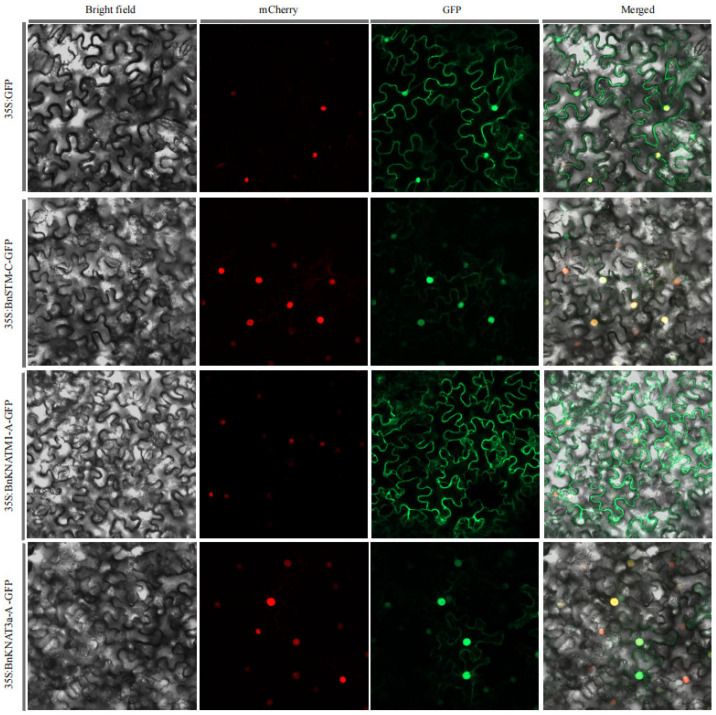
Sub-cellular localization of empty vector and three KNOX-GFP proteins. The empty vector and pART27-KNOX vectors were transformed into tobacco leaves, respectively, by using Agrobacterium tumefaciens mediated method. Three days later, the GFP (green color) and RFP (red color) fluorescence signals were observed by confocal microscopy.

The subcellular localization of the BnKNOX proteins was initially predicted to be nuclear using Cell-PLoc 2.0. However, the experimental results showed that BnSTM-C and BnKNAT3a-A were predominantly localized to the nucleus, while BnKNATM1-A was found in the cytoplasm. In contrast, GFP alone exhibited a diffuse pattern throughout the entire cell (Figure 9).

## 3. Discussion

### 3.1. Conservation and Evolution of KNOX Proteins in Plants

Genome evolutionary events and developmental biology reveal historical relationships, genome contractions and expansions, the evolution of functions and genome architecture [52]. *KNOX* genes represent a subfamily within the homeobox gene family, which is a key regulator of cell fate and body plan specification at the early stages of embryogenesis in higher organisms [53]. Screening and classification of KNOX proteins from lower to higher plants have been conducted on the basis of sequence conservation and domain organization. *KNOX* genes have been divided into Class I and Class II on the basis of sequences and expression patterns [8].

Based on the domain organization of KNOX1 and KNOX2, it is evident that KNOX genes are relatively recent additions to the plant kingdom, with their presence limited to higher plants (from moss to flowering plants). Even the homeobox gene family is ancient and conserved with their copies increasing during evolution (Figure 2; Supplement File S1). The classification of KNOX proteins into three classes (Figure 2). Class I and Class II are similar to the previous results [8]. AtKNATM, a novel KNOX member lacking a homeodomain domain [17] is classified as Class III (Class KNATM) and exclusively found in dicots. These findings are consistent with previous studies (Figure 2) [39]. Class II is highly conserved and present in all higher plants except Group II, which is restricted to Cruciferae. On the other hand, Class I and Class III are specific to vascular plants or dicots (Figure 2). Domain organization analysis shows that Class I/II carry conserved KNOX1, KNOX2, ELK and homeobox domains (Figure 3 and Figure 4), while Class III lacks the latter two domains (Figure 5). The differentiation of *KNOX* genes occurred during the evolutionary process of organisms. It is likely that Class I and Class II emerged after the divergence between higher/lower plants, whereas Class III appeared following the separation of dicots/monocots (Figure 2).

### 3.2. KNOX Orthologs Among B. napus, B. rapa and B. oleracea

The *Brassica* species, including *B. napus*, *B. rapa*, and *B. oleracea*, are economically important crops and serve as model plants for studying gene duplication events. These species are closely related to *Arabidopsis* and have been extensively sequenced in genomics research. Genomics has undergone large-scale gene silencing or elimination events after the divergence of the *Arabidopsis* and *Brassica* lineages. A chromosome duplication event might have occurred between the evolution of *Arabidopsis* and *B. rapa*. The diploid *B. rapa* genome has undergone genome triplication and lost genes [43,54,55]. The diploid *B. oleracea* genome has been actively rearranged since the divergence from *Arabidopsis*, potentially due to polyploidization [56,57]. The duplication of *KNOX* genes occurs after the separation of *Brassica* from *Arabidopsis* (Supplement Files S1 and S2). The number of *KNOX* genes in *Brassica* (15 in *B. rapa* and 14 in *B. oleracea*) is less than twice the number in *Arabidopsis* (9). Whole genomic duplication may have included *KNOX* gene duplications as most homologous genes in Brassica map to different chromosomes. They have also undergone large-scale gene silencing or elimination events after the divergence of the *Arabidopsis* and *Brassica* lineages. Synteny information and mutually best hit approach can be used to identify orthologous gene pairs, which tend to keep their original functions among different genomes [58]. The homologous *KNOX* genes of *B. rapa* and *B. oleracea* fall into the same clades in the phylogenetic tree and are all syntenic with corresponding *Arabidopsis* genes. Hence, these *KNOX* genes are orthologs (Figure 2, Figure 3, Figure 4 and Figure 5; Supplement File S2). Allopolyploid *B. napus* includes 32 *KNOX* genes, three more than the combined total of the *KNOX* genes in *B. rapa* and *B. oleracea* (Supplement Files S2 and S3). Compared with *Arabidopsis*, *B. napus* has six nonsyntenic genes, namely, *BnKNAT7a-A/1b-C/4b-C* and *BnKNAT6a-C/7a-C/7c-C*. The former exhibit mutual best hits with *BrKNAT7a* and *BoKNAT1/4b* along with their respective orthologous genes, while it is possible that *BnKNAT6a-C/7a-C/7c-C* represent paralogous duplicates generated after *B. napus* formation (Supplement Files S2 and S3).

The subgenomes of *B. napus* exhibit a lower frequency of homeologous exchange [59]. The homologs of *B. rapa* and *B. napus* can be Reciprocal Best BLASTp except *BnKNAT3a-A*. While all KNOX proteins from *B. oleracea* show significant hits against the homologs of *B. rapa*, five proteins from *B. rapa* (BrSTM, BrKNAT6a/6b/7a/7b) fail to return to corresponding targets of *B. oleracea*. Similar events are observed between *B. oleracea* and the C- genome of *B. napus* (Supplement File S3). These findings suggest that *KNOX* homeologous exchange events may occur more frequently in the C-genome than in the A-genome. Furthermore, we observe that BoKNAT1/3a/4b best hits the A-genome homologs of *B. napus* and that BrKNAT6b best hits the C- genome homologs BnKNAT6b-C (Supplement File S3). The function of a few *Brassica* KNOX orthologs might be high of conservation. The orthologs of the homeobox gene BREVIPEDICELLUS/KNAT1 in three *Brassica* species are conserved at the nucleotide and amino acid levels, and BnKNAT1 complements the *Arabidopsis* bp mutation [60].

### 3.3. KNOX Gene Duplication and Diversity in B. napus, B. rapa and B. oleracea

Gene duplications increase molecular diversity and serve as a foundation for organismal evolution. High-frequency polyploidy plays a crucial role in plant evolution. Although major chromosomal rearrangements have not been observed, homeologous recombination events between A- and C-genomes happen commonly [61]. *B. rapa* has duplicated *BrKNAT3/4/5/6/7*, whereas *B. oleracea* has duplicated *BoKNAT3/4/5/6*. The syntenic analysis of *KNOX* genes between *Arabidopsis* and *Brassica* species shows synteny between *BrKNAT3a/3b/BoKNAT3a/3b* and *AtKNAT3*, *BrKNAT4a/4b/BoKNAT4a/4b* and *AtKNAT4*, *BrKNAT5a/5b/BoKNAT5a/5b* and *AtKNAT5*, *BrKNAT6a/6b/BoKNAT6a/6b* and *AtKNAT6*, and *BrKNAT7a/7b* and *AtKNAT7* (Supplement File S2). We speculate that the duplication of BrKNAT7a/7b may be due to segmental rearrangement since it is mapped to chromosome A09. Others may result from genomic duplication. The confirmation of four genes in *B. oleracea* remains uncertain due to the lack of clarity regarding their physical mapping on the chromosomes. Pseudogenization, conservation of gene function, subfunctionalization and neofunctionalization are recognized as the main evolutionary fates of duplicated genes [62]. Neofunctionalization and subfunctionalization have been proposed as important processes driving the retention of duplicated genes [63]. Many mechanisms exist to silence or eliminate the duplicated genes [64].

Shifts of domain organization and expression patterns suggest the differentiation of functions. We find that most of the KNOX proteins are conserved, but several homologous genes have or lose some domains relative to the donors. Both *Brassica* species have two conserved copies of KNAT3 and KNAT5, one conserved copy of KNAT6 and two differentiated copies of KNATM. In addition, *B. rapa* has two conserved copies of BrKNAT4 and BrKNAT7, while *B. oleracea* shows conservation for BoKNAT4 but divergence for BoKNAT6 on the contrary.

BnKNAT1a-A/BnKNAT5a-A/5a-C (Figure 3), and BnKNATM2-A (Figure 5) have EBV-NA3, Virul-Fac and KNOX1 domains in comparison to BrKNAT1a/BrKNAT5a/BoKNAT5a (Figure 3) and BrKNATM2 (Figure 5), respectively. BnKNAT4a-C (Figure 4) lacks ELK and Nit-Regul-home domains, while BnKNATM1-A/1-C (Figure 5) lacks ChlamPMP_M domain relative to BoKNAT4a (Figure 4) and BrKNATM1/BoKNATM1 (Figure 5), correspondingly. In addition, BnKNAT1a-C (Figure 3) loses the KNOX1 domain, but BnKNAT7b-C/7c-C (Figure 4) only carry the KNOX1 domain. The majority of duplicated genes in allopolyploids evolve independently, and their functional diversification is a prominent feature of the long-term evolution of polyploids [65]. Changes in domain organizations suggest that KNOX genes also evolve and that the functions of these homologous genes may be differentiated in *B. napus*. Several members undergo pseudogenization, whereas others experience neofunctionalization or subfunctionalization.

### 3.4. Potential Roles of BnKNOX Genes Related to Plant Growth and Development

During our research, the analysis of transcriptomic data for 32 *BnKNOX* genes has served as a crucial foundation for conducting functional analysis. The qRT-PCR results of this study complement and confirm the transcriptome data. According to the qRT-PCR results, significant variations in the expression profiles of 32 *BnKNOX* transcripts were observed across different reproductive tissues. These differences were found to be closely associated with specific cis-elements identified in the promoter region of *BnKNOXs*, including auxin-responsive elements, light-responsive elements, circadian control elements, and hormone response elements. Class I *KNOX* genes are reported mainly expressed in meristem and regulating the transcription [28]. *BnSTM-A* and *BnSTM-C* share resembling *cis*-elements, gene structures and expression patterns, suggesting that they may have similar functions in plant development. Similar to the expression pattern of Class I genes that *AtSTM/AtKNAT1/AtKNAT2/KNAT6* were highly expressed in inflorescence tissues in Arabidopsis, the genes of Class I in *B. napus* were highly expressed in different development stages of Buds. Previous studies have shown that Class II *KNOX* genes *AtKNAT3/AtKNAT7* play an essential role in mucilage production in the early development stage of Arabidopsis seeds. Interestingly, our findings indicate that the BnKNOX genes in Class III (*BnKNATM1-A/1-C*, *BnKNATM2-A/2-C*) exhibited pronounced expression patterns in seeds, suggesting species-specific and diverse gene functions. Moreover, the high expression levels of *BnKNAT7a-A/a-C/7b-A/7b-C/7c-C* in stigma suggest their potential involvement in regulating female gametophytes morphogenesis. Additionally, the expression patterns of duplicated gene pairs (Supplement File S5) might differ from that of the genes in their subgroups. For example, *BnKNAT3a-A is* mainly expressed in all floral tissues except stigmas and seeds while *BnKNAT4a-A* is expressed only in the development stage of buds. *BnKNAT7a-A* and *BnKNAT7c-C* showed high expression in anther and stamen. These findings indicate that gene duplication could potentially drive structural diversity and result in functional disproportionation or redundancy. The subcellular localization studies lend weight to the possibility that distinct gene subfamilies may have experienced functional divergence.

Overall, these findings provide valuable insights for further investigating the role of these genes in plant morphogenesis regulation and adaptation to environmental changes.

## 4. Materials and Methods

### 4.1. KNOX Protein Identification and Chromosome Map Construction

Sequences of *Arabidopsis* KNOX proteins, namely, AtSTM (AT1G62360), AtKNAT1 (AT4G08150), AtKNAT2 (AT1G70510), AtKNAT6 (AT1G23380), AtKNAT3 (AT5G25220), AtKNAT4 (AT5G11060), AtKNAT5 (AT4G32040), AtKNAT7 (AT1G62990) and AtKNATM (AT1G14760), were used as queries to search target proteins from the Brassica website (http://brassicadb.cn, Version 3.0) and the Phytozome (http://www.phytozome.net/, version 13.0). The recovered protein sequences were then used as queries in Blastp in TAIR (TAIR—BLAST (arabidopsis.org)), and the domains of sequences with hits to relevant KNOX proteins in Arabidopsis were analyzed. The gene locus information of *B. rapa*, *B. oleracea* and *B. napus* was used to generate chromosome maps by using the MapChart 2.2 program [66] (Voorrips, 2002). A syntenic analysis of KNOX genes in the Arabidopsis and Brassica species was conducted on the website (http://brassicadb.cn, Version 3.0).

### 4.2. Analysis of Protein Domain Organization

The domain organization of protein sequences was analyzed using NCBI-CD searches of the Pfam database (NCBI Conserved Domain Search) [67]. The low-complexity filter was turned off, the Expect Value was set at 10 to detect short domains or regions of minimal conservation and domains were verified and named according to the SMART database (SMART: Main page (embl-heidelberg.de)).

### 4.3. Phylogenetic Analysis

Multiple sequence alignments were performed using the Clustal W program [68]. The resulting file was subjected to a phylogenic analysis using the MEGA 11.0 program [69]. Trees were constructed on the basis of the protein sequences using Neighbor-Joining methods with parameters (Pairwise deletion option; p-distance substitution model; and 1000 Bootstrap test). The duplicated genes and homologs of Brassica were confirmed via the phylogenetic tree and syntenic analysis (BRAD (brassicadb.cn)).

### 4.4. Analysis of Cis-Acting Elements of BnKNOXs Promoters and Gene Structures in B. napus

For the investigation of *cis*-acting elements with *BnKNOXs*, the 2000 bp upstream gene sequences were extracted by TBtools [70], and then the cis-acting elements were identified by PlantCARE database (http://bioinformatics.psb.ugent.be/webtools/plantcare/html/). *Cis*-acting element numbers and annotation were visualized using TBtools (V.2.315). To further explore the characteristics of *BnKNOXs*, exon-intron structure was analyzed using the Gene Structure Display Server and gene structures were visualized by TBtools.

### 4.5. Collinearity Analysis

The collinearity relationship of the *BnKNOXs* was detected by TBtools. The results were visualized by Advanced Circos. Based on the full-length-CDS sequence covering and identity of amino acid detected by Blastn/Blastp in NCBI, the non-synonymous substitution rate (Ka) and synonymous substitution rate (Ks) value of the duplicated gene pairs were calculated by TBtools. The ratio of Ka/Ks was used to estimate the mode of selection and considered positive, negative or neutral selection when Ka/Ks ratio was >1, <1 or =1, respectively [71,72]. Divergence time (million years ago, Mya) was estimated by using a Simple Ka/ Ks Calculator tool on TBtools software [73].

### 4.6. Expression Levels of BnKNOXs in Different Tissues

The expression files of *BnKNOXs* in various developmental stages (Bolting, Initial flowering, Podding and maturation) from the cultivar XiangYou15 (XY15) of *B. napus* were obtained through *BrassicaEDB* (BrassicaEDB - A Gene Expression Database for Brassica Crops). The log_2_FPKM (Fragments Per Kilobase of exon model per Million mapped fragments) values were used to evaluate gene expression in different tissues and were showed via TBtools.

### 4.7. Validation of Expression Levels of BnKNOXs by qRT-PCR

To determine the expression levels of *BnKNOXs*, total RNA was isolated from various tissues following the protocol of RNA isolater Total RNA Extraction Reagent. The obtained RNA (1 μg) was used as the template in synthesizing cDNA for qRT-PCR using the Hiscript III RT SuperMix (Vazyme, Nanjing, China). The qRT-PCR was completed using three technical and biological replicates. The relative expression levels were calculated by the 2^−ΔΔCt^ method. In this study, to ensure the accuracy and reliability of gene expression analysis, two reference genes, *PPR* (Postsynaptic protein-related) and *GDI1* (Guanosine nucleotide diphosphate dissociation inhibitor 1), were selected [74]. The geomean value of these reference genes was used to recalculate the ΔΔCt. The relative expression data were analyzed and shown using TBtools.

### 4.8. Subcellular Localization of the BnKNOX::GFP Fusion Protein

To determine the specific subcellular localization of the proteins encoded by the genes of interest, we selected one representative candidate gene from each of the three classes: BnSTM-C (Class I), BnKNATM1-A (Class II), and BnKNAT3a-A (Class III). The corresponding genes were amplified and inserted into the pART27 vector in-frame with GFP through homologous recombination. The constructs were introduced into Agrobacterium tumefaciens GV3101, which was then infiltrated into tobacco epidermal cells for transient expression in tobacco (*Nicotiana tabacum* L.) leaves. pART27 alone was used as control. After 3 days of infiltration, GFP fluorescence was visualized using scanning confocal laser microscopy. In this study, mCherry served as the nuclear localization marker (Red fluorescence).

## 5. Conclusions

Plant KNOX proteins can be divided into three classes. Class I and Class III are exclusively found in vascular plants or dicots, respectively, whereas Class II is the most conserved and found in all higher plants. *B. napus* and its parental species *B. rapa* and *B. oleracea* possess 15, 14 and 32 KNOX genes, respectively. During allotetraploid formation, *B. napus* shares most highly conserved *KNOX* genes with their A- or C- genome contributors. *B. napus* eliminates some KNOX orthologs from its parents, and forms new homologs due to gene duplication. The divergence of *KNOX* gene functions occurs due to shifts observed in a few duplicated genes or orthologous proteins between *B. napus* and its parental species *B. rapa* and *B. olerecea* during allotetraploid formation process. The *KNOX* gene evolution and function diversity during allotetraploid formation are crucial issues. The evolutionary relationship, gene structure, *cis*-acting elements, expression patterns and subcellular localization further indicate that members of the *BnKNOX* gene family exhibit both conservative characteristics as well as diversity. The results from sub-cellular localization studies strengthen the hypothesis that different subfamilies of genes have undergone functional differentiation. These findings provide fundamental insights into understanding the role of KNOXs in growth and development processes specific to *Brassica napus*.

## Data Availability

The original contributions presented in this study are included in the article/Supplementary Materials. Further inquiries can be directed to the corresponding author.

## Supplementary Materials

The following supporting information can be downloaded at: https://www.mdpi.com/article/10.3390/plants14142167/s1. Supplement File S1. KNOX homologs of plant species. Supplement File S2. Syntenic analysis of KNOX homologs from Arabidopsis and three *Brassica* species. Supplement File S3. Reciprocal Blastp analysis of KNOX homologs from three *Brassica* species. Supplement File S4. KNOX protein sequences in Class I. Supplement File S5. KNOX protein sequences in Class II. Supplement File S6. KNOX protein sequences in Class III. Supplement File S7. Information of cis-acting elements on the promoters of KNOX genes in *B. napus.* Supplement File S8. Collinear gene pairs in *B. napus.* Supplement File S9. Duplication genes of KNOX in *B. napus.* Supplement File S10. Detailed dates of RNA-seq and qRT-PCR. Supplement File S11. Primers list of KNOX genes in *B. napus.*
