# Supplementary material for: Genome-Wide Analysis of KNOX Genes: Identification, Evolution, Comparative Genomics, Expression Dynamics, and Sub-Cellular Localization in Brassica napus
Source: Plants (Basel). 2025 Jul 14;14(14):2167. doi: 10.3390/plants14142167 (PMC12300509; doi:10.3390/plants14142167)
Supplement: Supplementary file 1 [file plants-14-02167-s001.zip › Supplement file S1 KNOX Proteins list used in this paper.pdf]

Supplement Table S1. KNOX homologs of plant species

Name	Locus	Genomic Length (bp)	CDS Length (bp)	Protein Length (aa)	No. of Introns
***Medicago truncatula* (7)**					
MtSTM	Medtr5g085860	3561	939	312	4
MtKNAT3a	Medtr3g106400	3836	1122	373	6
MtKNAT3b	Medtr4g116730	4958	999	332	4
MtKNAT6a	Medtr5g033720	8331	951	316	5
MtKNAT6b	Medtr1g084060	6340	807	268	5
MtKANT7	Medtr5g011070	3643	879	292	6
MtKNATM	Medtr6g071190	3784	486	161	3
***Populus trichocarpa* (16)**					
PtSTM1	Potri.004G004700	4369	1110	369	4
PtSTM2	Potri.011G011100	4373	1122	373	4
PtKANT1	Potri.002G113300	6341	1107	368	5
PtKANT3a	Potri.006G259400	5145	1275	424	6
PtKANT3b	Potri.018G022700	5133	1278	425	6
PtKNAT3c	Potri.006G190000	5504	1017	338	5
PtKNAT3d	Potri.018G114100	4420	1005	334	5
PtKANT6a	Potri.008G188700	8346	1026	341	5
PtKANT6b	Potri.010G043500	10307	930	309	5
PtKANT6c	Potri.012G087100	8044	1023	340	5
PtKANT6d	Potri.015G079100	8001	1044	347	5
PtKANT6e	Potri.005G017200	4687	951	316	5
PtKANT6f	Potri.005G014200	5189	954	317	5
PtKANT6g	Potri.013G008600	4664	963	320	5
PtKNAT7	Potri.001G112200	5065	906	301	5
PtKNATM	Potri.012G043400	4910	546	181	5
***Fragaria vesca* (8)**					
FvSTM1	19507-v1.0-hybrid	6135	1413	470	7
FvSTM2	30482-v1.0-hybrid	2983	759	252	3
FvKANT1	02647-v1.0-hybrid	4369	1434	477	7
FvKNAT3	30834-v1.0-hybrid	1781	972	323	6
FvKNAT6a	03606-v1.0-hybrid	6121	1176	391	6
FvKNAT6b	31590-v1.0-hybrid	2775	990	330	5
FvKANT7	32356-v1.0-hybrid	2288	807	289	5
FvKNATM	09344.1-v1.0-hybrid	5005	957	318	7
***Arabidopsis thaliana* (9)**					
AtSTM	AT1G62360	3141	1149	382	4
AtKNAT1	AT4G08150	3267	1197	398	5
AtKNAT2	AT1G70510	5946	933	310	5
AtKNAT3	AT5G25220	2511	1296	431	6
AtKNAT4	AT5G11060	3188	1182	393	6
AtKNAT5	AT4G32040	2292	1152	383	6
AtKNAT6	AT1G23380	5361	984	327	5
AtKNAT7	AT1G62990	3324	876	291	5
AtKNATM	AT1G14760	562	429	142	2
***Brassica rapa* (15)**					
BrSTM	Bra027050	2882	1149	382	4

BrKNAT1	Bra000638	3681	1164	387	5
BrKNAT2	Bra007920	5242	984	327	5
BrKNAT3a	Bra009826	1729	1245	414	6
BrKNAT3b	Bra020491	2076	1182	393	5
BrKNAT4a	Bra006085	2442	1161	386	7
BrKNAT4b	Bra028554	3306	1164	387	5
BrKNAT5a	Bra011317	1889	1125	374	5
BrKNAT5b	Bra023965	1586	1152	383	6
BrKNAT6a	Bra024593	1587	630	209	4
BrKNAT6b	Bra016348	5229	945	314	5
BrKNAT7a	Bra036636	3127	885	294	5
BrKNAT7b	Bra027006	6062	888	295	5
BrKNATM1	Bra026190	676	420	139	3
BrKNATM2	Bra026805	695	408	135	3
***Brassica Oleracea* (14)**					
BoSTM	Bol011007	2925	1149	249	4
BoKNAT1	Bol015104	3678	1158	385	5
BoKNAT2	Bol035035	5819	984	214	5
BoKNAT3a	Bol022368	1736	1257	418	6
BoKNAT3b	Bol016412	1874	1194	397	5
BoKNAT4a	Bol009058	2428	1236	411	6
BoKNAT4b	Bol024692	2211	1191	396	5
BoKNAT5a	Bol017942	1859	1125	374	5
BoKNAT5b	Bol033762	1571	1203	400	6
BoKNAT6a	Bol023484	1214	666	221	4
BoKNAT6b	Bol001002	5893	945	314	5
BoKNAT7	Bol029705	3765	885	294	5
BoKNATM1	Bol038072	684	414	137	3
BoKNATM2	Bol031520	678	408	135	3
***Brassica napus* (32)**					
BnSTM-A	BnaA09g13310D	2992	1152	383	4
BnSTM-C	BnaC09g13580D	3039	1152	383	4
BnKNAT1-A	BnaA03g23610D	3657	1209	402	6
BnKNAT1a-C	BnaC03g28030D	4148	582	193	3
BnKNAT1b-C	BnaCnng59830D	1780	1050	349	5
BnKNAT2-A	BnaA02g14950D	5187	984	327	5
BnKNAT2-C	BnaC02g19900D	5913	984	327	6
BnKNAT3a-A	BnaA06g27560D	1801	900	299	6
BnKNAT3a-C	BnaC07g29530D	5135	1335	444	7
BnKNAT3b-A	BnaA02g32110D	2530	1263	420	6
BnKNAT3b-C	BnaC02g40790D	2708	1215	404	6
BnKNAT4a-A	BnaA02g00810D	2808	1161	386	6
BnKNAT4a-C	BnaCnng20070D	2628	909	302	6
BnKNAT4b-A	BnaA03g03190D	2807	1182	393	6
BnKNAT4b-C	BnaC03g04580D	2519	1182	393	6
BnKNAT5a-A	BnaA01g04870D	2293	1125	374	5
BnKNAT5a-C	BnaC01g06410D	2321	1125	374	5
BnKNAT5b-A	BnaA03g51900D	1752	1149	382	6
BnKNAT5b-C	BnaC07g43650D	1957	1203	400	6

BnKNAT6a-A	BnaA09g31100D	1588	708	235	5
BnKNAT6a-C	BnaC08g06320D	1215	666	221	4
BnKNAT6b-A	BnaA08g20500D	1749	627	208	4
BnKNAT6b-C	BnaC05g18670D	5978	945	314	5
BnKNAT7a-A	BnaA09g12980D	3728	885	294	5
BnKNAT7a-C	BnaC04g20090D	3611	885	294	5
BnKNAT7b-A	BnaA09g52990D	4239	882	293	5
BnKNAT7b-C	BnaCnng51440D	381	315	104	2
BnKNAT7c-C	BnaC09g12900D	309	309	102	1
BnKNATM1-A	BnaA09g45470D	850	426	141	3
BnKNATM2-A	BnaA06g09570D	694	408	135	3
BnKNATM1-C	BnaC05g10940D	678	408	135	3
BnKNATM2-C	BnaC08g39310D	874	414	137	3
***Zea mays* (16)**					
ZmKNAT1a	GRMZM2G060050	1023	549	182	1
ZmKNAT1b	GRMZM2G017087	7721	1080	359	7
ZmKNAT1c	GRMZM2G135447	6986	1095	364	5
ZmKNAT1d	GRMZM2G028041	6194	1056	351	5
ZmKNAT1e	GRMZM2G452178	6270	1083	360	5
ZmKNAT1f	GRMZM2G000743	1357	582	193	3
ZmKNAT1g	GRMZM2G002225	22510	987	328	5
ZmKNAT1h	GRMZM2G061101	6697	1059	352	5
ZmKNAT3a	GRMZM2G055243	8398	951	316	6
ZmKNAT3b	GRMZM2G370332	3707	903	300	5
ZmKNAT3c	GRMZM2G433591	3455	987	328	5
ZmKNAT6a	GRMZM2G087741	9840	888	295	5
ZmKNAT6b	GRMZM2G094241	5567	924	307	5
ZmKNAT6c	GRMZM5G832409	11481	897	298	5
ZmKNAT7a	GRMZM2G060507	3704	576	191	5
ZmKNAT7b	GRMZM2G159431	4639	933	310	5
***Oryza sativa* (13)**					
OsKNAT1a	Os03g51690	10585	1086	361	5
OsKNAT1b	Os03g56110	5964	1026	341	5
OsKNAT1c	Os03g56140	5078	1158	385	4
OsKNAT1d	Os07g03770	6024	1068	355	5
OsKNAT1e	Os03g51710	6460	1134	377	4
OsKNAT1f	Os03g47022	925	501	166	4
OsKNAT1g	Os03g47042	1274	585	194	3
OsKNAT3a	Os08g19650	6566	837	278	5
OsKNAT3b	Os06g43860	3348	972	323	6
OsKNAT3c	Os02g08544	3471	906	301	6
OsKNAT6a	Os01g19694	7505	906	301	5
OsKNAT6b	Os05g03884	6492	936	311	5
OsKNAT7	Os03g03164	5107	945	314	5
***Brachypodium distachyon* (11)**					
BdKNAT1a	Bradi1g10047	6951	1119	372	5
BdKNAT1b	Bradi1g07247	3569	1038	345	5
BdKNAT1c	Bradi1g57607	4752	966	321	5
BdKNAT1d	Bradi1g12677	1682	756	251	5

BdKNAT1e	Bradi1g12690	6347	942	313	5
BdKNAT3a	Bradi3g19927	5597	1107	368	6
BdKNAT3b	Bradi1g30730	3585	918	305	6
BdKNAT3c	Bradi3g06170	3644	951	316	6
BdKNAT6a	Bradi2g11540	6353	873	290	5
BdKNAT6b	Bradi2g38390	5227	903	300	5
BdKNAT7	Bradi1g76970	5233	945	314	5
***Physcomitrella patens* (4)**					
PpKNAT2a	Pp1s33_357V6	3304	1308	445	6
PpKNAT2b	Pp1s235_27V6	3979	1527	508	7
PpKNAT3a	Pp1s77_59V6	3005	1557	518	6
PpKNAT3b	Pp1s154_83V6	6569	1911	636	11
***Selaginella moellendorffii* (4)**					
SmKNAT1a	159366	3071	1242	413	6
SmKNAT1b	415291	2242	1011	336	6
SmKNAT3a	90744	1761	882	293	5
SmKNAT3b	135843	1076	828	275	5
Ostreococcus lucimarinus					
OlKNAT7	gwEuk.4.361.1	732	732	236	1
Phaeodactylum tricornutum					
	Phatr2|55150#	2667	1221	406	3
Chlamydomonas reinhardtii					
	Cre08.g375400#	6446	2805	934	7
Volvox carteri					
	Vocar20012185m#	1918	342	113	4
#: They are belonged into HOX gene family but lack of any KNOX domain.					
