# Supplementary material for: Genome-Wide Analysis of KNOX Genes: Identification, Evolution, Comparative Genomics, Expression Dynamics, and Sub-Cellular Localization in Brassica napus
Source: Plants (Basel). 2025 Jul 14;14(14):2167. doi: 10.3390/plants14142167 (PMC12300509; doi:10.3390/plants14142167)
Supplement: Supplementary file 1 [file plants-14-02167-s001.zip › Supplement file S2 Reciprocal Blastp analysis.pdf]

Table S2. Reciprocal Blastp analysis of KNOX homologs from three *Brassica* species

Name	Locus	Reciprocal Blastp 1	Reciprocal Blastp 2	Reciprocal Blastp 3
Brassica rapa (15)				
BrSTM	Bra027050		BoKNAT1	BnSTM-A
BrKNAT1	Bra000638		BoKNAT1	BnKNAT1-A
BrKNAT2	Bra007920		BoKNAT2	BnKNAT2-A
BrKNAT3a	Bra009826		BoKNAT3a	BnKNAT3b-A
BrKNAT3b	Bra020491		BoKNAT3b	BnKNAT3b-A
BrKNAT4a	Bra006085		BoKNAT4b	BnKNAT4a-A
BrKNAT4b	Bra028554		BoKNAT4b	BnKNAT4b-A
BrKNAT5a	Bra011317		BoKNAT5a	BnKNAT5a-A
BrKNAT5b	Bra023965		BoKNAT5b	BnKNAT5b-A
BrKNAT6a	Bra024593		BoKNAT2	BnKNAT6a-A
BrKNAT6b	Bra016348		BoKNAT2	BnKNAT6b-C
BrKNAT7a	Bra036636		BoKNAT3a	BnKNAT7a-A
BrKNAT7b	Bra027006		BoKNAT3a	BnKNAT7b-A
BrKNATM1	Bra026190		BoKNATM1	BnKNATM1-A
BrKNATM2	Bra026805		BoKNATM2	BnKNATM2-A
Brassica Oleracea (14)				
BoSTM	Bol011007	BrSTM		BnSTM-C
BoKNAT1	Bol015104	BrKNAT1		BnKNAT1-A
BoKNAT2	Bol035035	BrKNAT2		BnKNAT2-C
BoKNAT3a	Bol022368	BrKNAT3a		BnKNAT3b-A
BoKNAT3b	Bol016412	BrKNAT3b		BnKNAT3b-C
BoKNAT4a	Bol009058	BrKNAT4a		BnKNAT4a-A
BoKNAT4b	Bol024692	BrKNAT4b		BnKNAT4b-C
BoKNAT5a	Bol017942	BrKNAT5a		BnKNAT5a-C
BoKNAT5b	Bol033762	BrKNAT5b		BnKNAT5b-C
BoKNAT6a	Bol023484	BrKNAT6a		BnKNAT6a-C
BoKNAT6b	Bol001002	BrKNAT6b		BnKNAT6b-C
BoKNAT7	Bol029705	BrKNAT7a		BnKNAT7a-C
BoKNATM1	Bol038072	BrKNATM1		BnKNATM2-C
BoKNATM2	Bol031520	BrKNATM2		BnKNATM1-C
***Brassica napus* (32)**				
BnSTM-A	BnaA09g13310D	BrSTM	BoKNAT1	BnSTM-C
BnSTM-C	BnaC09g13580D	BrSTM	BoKNAT1	BnSTM-A
BnKNAT1-A	BnaA03g23610D	BrKNAT1	BoKNAT1	BnKNAT1b-C
BnKNAT1a-C	BnaC03g28030D	BrKNAT1	BoKNAT1	BnKNAT1-A
BnKNAT1b-C	BnaCnng59830D	BrKNAT1	BoKNAT1	BnKNAT1-A
BnKNAT2-A	BnaA02g14950D	BrKNAT2	BoKNAT2	BnKNAT2-C
BnKNAT2-C	BnaC02g19900D	BrKNAT2	BoKNAT2	BnKNAT2-A
BnKNAT3a-A	BnaA06g27560D	BrKNAT4a	BoKNAT3a	BnKNAT3a-C
BnKNAT3a-C	BnaC07g29530D	BrKNAT4a	BoKNAT3a	BnKNAT3a-A
BnKNAT3b-A	BnaA02g32110D	BrKNAT3b	BoKNAT3b	BnKNAT3b-C
BnKNAT3b-C	BnaC02g40790D	BrKNAT3a	BoKNAT3b	BnKNAT3b-A
BnKNAT4a-A	BnaA02g00810D	BrKNAT4a	BoKNAT4b	BnKNAT4b-A
BnKNAT4a-C	BnaCnng20070D	BrKNAT4a	BoKNAT4b	BnKNAT4a-A
BnKNAT4b-A	BnaA03g03190D	BrKNAT4b	BoKNAT4b	BnKNAT4b-C
BnKNAT4b-C	BnaC03g04580D	BrKNAT4b	BoKNAT4b	BnKNAT4b-A

BnKNAT5a-A	BnaA01g04870D	BrKNAT5a	BoKNAT5a	BnKNAT5a-C
BnKNAT5a-C	BnaC01g06410D	BrKNAT5a	BoKNAT5a	BnKNAT5a-A
BnKNAT5b-A	BnaA03g51900D	BrKNAT5b	BoKNAT5b	BnKNAT5b-C
BnKNAT5b-C	BnaC07g43650D	BrKNAT5b	BoKNAT5b	BnKNAT5b-A
BnKNAT6a-A	BnaA09g31100D	BrKNAT6a	BoKNAT2	BnKNAT6a-C
BnKNAT6a-C	BnaC08g06320D	BrKNAT6a	BoKNAT2	BnKNAT6a-A
BnKNAT6b-A	BnaA08g20500D	BrKNAT6b	BoKNAT2	BnKNAT6b-C
BnKNAT6b-C	BnaC05g18670D	BrKNAT6b	BoKNAT2	BnKNAT2-C
BnKNAT7a-A	BnaA09g12980D	BrKNAT7a	BoKNAT3a	BnKNAT7a-C
BnKNAT7a-C	BnaC04g20090D	BrKNAT7a	BoKNAT3b	BnKNAT7a-A
BnKNAT7b-A	BnaA09g52990D	BrKNAT7b	BoKNAT3a	BnKNAT7a-A
BnKNAT7b-C	BnaCnng51440D	BrKNAT7a	BoKNAT5b	BnKNAT7a-C
BnKNAT7c-C	BnaC09g12900D	BrKNAT7a	BoKNAT3a	BnKNAT7a-C
BnKNATM1-A	BnaA09g45470D	BrKNATM1	BoKNATM1	BnKNATM2-C
BnKNATM2-A	BnaA06g09570D	BoKNATM2	BoKNATM2	BnKNATM1-C
BnKNATM1-C	BnaC05g10940D	BrKNATM2	BoKNATM2	BnKNATM2-A
BnKNATM2-C	BnaC08g39310D	BrKNATM1	BoKNATM1	BnKNATM1-A

Reciprocal Blastp 1: blastp between *Brassica napus/Brassica oleracea* and *Brassica rapa*;

Reciprocal Blastp 2: blastp between *Brassica napus/Brassica rapa* and *Brassica oleracea*;

Reciprocal Blastp 3: blastp between *Brassica rapa/Brassica oleracea* and *Brassica napus. Blastp*

Table S3. Syntenic analysis of *KNOX* homologs from Arabidopsis and three *Brassica* species

A. thaliana	***B. rapa* (15)**	***B. Oleracea* (14)**	***B. napus* (32)**
AtSTM	*BrSTM*	*BoSTM*	*BnSTM-A, BnSTM-C*
AtKNAT1	*BrKNAT1*	*BoKNAT1*	*BnKNAT1-A, BnKNAT1a-C*
/	/	/	*BnKNAT1b-C*
AtKNAT2	*BrKNAT2*	*BoKNAT2*	*BnKNAT2-A, BnKNAT2-C*
AtKNAT3	*BrKNAT3a, BrKNAT3b*	*BoKNAT3a,*	*BnKNAT3a-A, BnKNAT3b-A, BnKNAT3a-C,*
AtKNAT4	*BrKNAT4a, BrKNAT4b*	*BoKNAT4a, BoKNAT4b*	*BnKNAT4a-A, BnKNAT4a-C, BnKNAT4b-A*
/	/	/	*BnKNAT4b-C*
AtKNAT5	*BrKNAT5a, BrKNAT5b*	*BoKNAT5a, BoKNAT5b*	*BnKNAT5a-A, BnKNAT5a-C, BnKNAT5b-A,*
AtKNAT6	*BrKNAT6a, BrKNAT6b*	*BoKNAT6a, BoKNAT6b*	*BnKNAT6a-A, BnKNAT6b-A, BnKNAT6b-C*
/	/	/	*BnKNAT6a-C*
AtKNAT7	*BrKNAT7a, BrKNAT7b*	*BoKNAT7*	*BnKNAT7b-A, BnKNAT7b-C*
/	/	/	*BnKNAT7a-A, BnKNAT7a-C, BnKNAT7c-C*
AtKNATM	*BrKNATM1,*	*BoKNATM1,*	*BnKNATM1-A, BnKNATM1-C, BnKNATM2-A,*
